# Performance and usability evaluation of three LDH-based malaria rapid diagnostic tests in Kédougou, Senegal

**DOI:** 10.1186/s13071-025-06914-9

**Published:** 2025-07-12

**Authors:** Babacar Souleymane Sambe, Stephanie Zobrist, William Sheahan, Divya Soni, Aissatou Diagne, Ibrahima Sarr, Arona Sabene Diatta, Serigne Ousmane Mbacke Diaw, Allison Golden, Hannah Slater, Ihn Kyung Jang, Nerie Roa, Sampa Pal, Fatoumata Diene Sarr, Joseph Faye, Inès Vigan-Womas, Yakou Dieye, Moustapha Cisse, Gonzalo J. Domingo, Makhtar Niang

**Affiliations:** 1 Institut Pasteur de Dakar, Dakar, Senegal; 2 PATH, Seattle, USA; 3 PATH, New Delhi, India; 4 PATH, Dakar, Senegal

**Keywords:** Malaria, Diagnosis, Rapid diagnostic test, Lactate dehydrogenase, Histidine rich protein 2

## Abstract

**Background:**

The emergence of *Plasmodium falciparum* (*Pf*) parasites with deleted histidine-rich protein 2 and 3 (*hrp2/hrp3*) genes threatens the performance of HRP2-based malaria rapid diagnostic tests (RDTs). RDTs targeting *Pf* lactate dehydrogenase (LDH) may address current product limitations and improve case management. The objective of this study was to evaluate the performance and usability of three LDH-based RDTs in febrile patients.

**Methods:**

A cross-sectional diagnostic accuracy study was conducted in Kédougou, Senegal. Capillary blood was tested using the SD BIOLINE Ag Pf RDT (Abbott Diagnostics Korea Inc., Republic of Korea) and three LDH-based RDTs (BIOCREDIT Malaria Ag Pf [pLDH], BIOCREDIT Pf [pLDH/HRPII] and BIOCREDIT Pf/Pv [pLDH/pLDH]; Rapigen Inc., Republic of Korea). Venous blood was collected and used to repeat the BIOCREDIT RDTs and conduct microscopy. Frozen venous specimens were tested using a reference PCR assay. A quantitative multiplex malaria antigen assay measured antigen concentration. RDT performance was determined and analyzed as a function of antigen concentration distribution. Usability of the *Pf*-only BIOCREDIT tests was evaluated using a questionnaire.

**Results:**

Of the 220 participants, 154 (70%) were *Pf*-positive by PCR. The Pf (pLDH/HRPII) test demonstrated the highest sensitivity at 78% (95% confidence interval [CI] 70.9–84.5%); specificity was 89% (95% CI 79.4–95.6%). All RDTs performed better than microscopy (53% sensitivity). Although the HRP2 line on the Pf (pLDH/HRPII) test was more sensitive than the SD BIOLINE HRP2-based test (71%, 95% CI 63.6–78.4%), the sensitivities of the PfLDH lines alone on all three BIOCREDIT tests (61–64%) were lower than that of the SD BIOLINE HRP2 test. RDTs performed better when compared to antigen concentration over PCR results. Improved sensitivity of the Pf (pLDH/HRPII) test was driven by the HRP2 line. Line intensity correlated with antigen concentration. Predicted sensitivity using the analytical limit of detection (LOD) was comparable to the observed sensitivity. RDTs demonstrated acceptable usability.

**Conclusions:**

Both HRP2 and LDH contributed to the sensitivity of the best-performing *Pf-*RDT. In populations such as this with low rates of *hrp2/hrp3* deletions, the PfLDH line alone cannot compensate for the performance of the HRP2 line, even with the improved PfLDH LOD of the BIOCREDIT tests. RDT analytical LODs can be used to predict performance in populations with known antigen concentrations.

**Graphical Abstract:**

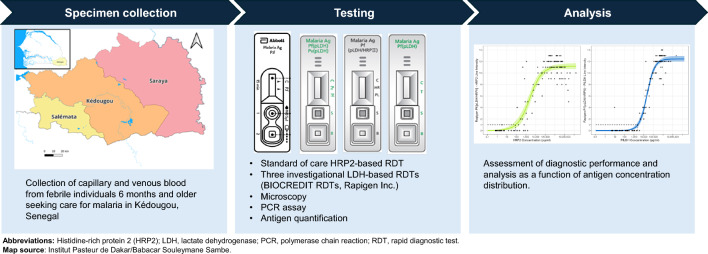

**Supplementary Information:**

The online version contains supplementary material available at 10.1186/s13071-025-06914-9.

## Background

Accurate and timely diagnosis of malaria is essential to ensure effective treatment for patients and accelerate control and elimination efforts. Lateral flow immunochromatographic rapid diagnostic tests (RDTs) for malaria are widely accepted in endemic settings due to their simplicity, low cost, minimal infrastructure requirements and rapid time to results [1, 2].

Malaria RDTs function by detecting specific protein antigens expressed by the malaria parasite in the blood of infected individuals [3]. Histidine-rich protein 2 (HRP2) and lactate dehydrogenase (LDH) are two common antigens targeted by malaria RDTs. The vast majority of RDTs used for the diagnosis of *Plasmodium falciparum* (*Pf*) malaria target HRP2, as this antigen is specific to *P. falciparum* and cannot be produced by other malarial species [4]. To date, HRP2 has been the preferred target for *Pf* RDTs due to its abundant production by the parasite, as well as greater heat stability and clinical sensitivity as compared to *Pf-*specific lactate dehydrogenase (PfLDH)-based RDTs [5–11]. Senegal, along with many other African countries where *Pf* is the predominant species [12, 13], currently relies primarily on HRP2-based RDTs for malaria diagnosis.

There are significant limitations to the widespread use of HRP2-based *Pf* RDTs. The HRP2 antigen can persist in the peripheral bloodstream for multiple weeks following parasite clearance and can, therefore, result in false-positive results among individuals who have recently received treatment. Most importantly, the performance of HRP2-based RDTs is threatened by increasing reports of parasites with deleted *hrp2/hrp3* genes [14, 15]. In settings with a significant prevalence of *hrp2/hrp3* gene deletions in symptomatic populations, the World Health Organization (WHO) advises switching to RDTs that do not rely exclusively on HRP2 detection [16]. Several studies have documented observations of *Pf hrp2/hrp3* gene deletions in Senegal [17, 18]. Recent modeling projects that deletions identified in Western Africa will increase [19] and, as such, further monitoring of deletion prevalence and its impact on RDT performance in this setting is warranted. A switch to PfLDH-based tests must take into account the significantly higher limits of detection (LOD) for LDH and, consequently, the lower clinical sensitivity of these tests [20]. For this reason, improvements in the performance of PfLDH-based tests are needed in order to minimize tradeoffs in sensitivity.

Rapigen Inc. (Suwon, Gyeonggi-do, Republic of Korea) has developed three novel, LDH-based malaria RDTs:

- The BIOCREDIT Malaria Ag Pf (pLDH/HRPII) RDT, with one test line for HRP2 and a separate test line for PfLDH
- The BIOCREDIT Malaria Ag Pf/Pv (pLDH/pLDH) RDT, with one test line for PfLDH and a second test line for *Plasmodium vivax* LDH (PvLDH)
- The BIOCREDIT Malaria Ag Pf (pLDH) RDT, with one test line for PfLDH.

Analytical benchmarking and clinical sensitivity modeling for these tests indicate that they have improved LODs for LDH that may result in higher clinical performance in terms of sensitivity toward *Pf* infections with *hrp2/hrp3* gene deletions and for *Pv* clinical infections [20]. Additionally, previous evaluations of these tests in Ghana, Burundi, Uganda, Djibouti and the Republic of Korea have also demonstrated promising clinical performance [21–25].

The aim of this study was to evaluate the clinical performance of these three PfLDH-based RDTs for malaria among a febrile population in Kédougou, Senegal, in comparison to two different reference methods: (i) a PCR assay and (ii) antigen concentration quantification by a chemiluminescent enzyme-linked immunosorbent assay (ELISA). The performance of microscopy and a currently available HRP2-based comparator RDT were also evaluated in the same population in order to enable informed decision-making regarding the recommendation of new, highly sensitive point-of-care tools for malaria.

## Methods

### Study design and population

Between November 2021 and February 2022, a cross-sectional diagnostic accuracy study of patients presenting with febrile symptoms was conducted in Kédougou, Senegal. The Kédougou region borders Guinea and Mali and its population experiences a high burden of *Pf* malaria with moderate seasonality [26, 27]. The 2020–2021 Senegal Malaria Indicator Survey (MIS) reported a cross-sectional household prevalence of 9.3% positivity for *Pf* malaria among children under the age of 5 years in Kédougou [28]. In the present study, patients aged 6 months and older presenting with febrile symptoms were recruited from five health facilities: the Kédougou Health Center, and the Tomboronkoto, Dalaba, Bandafassi, and Bantako health posts. Individuals weighing < 8 kg or those who had a serious illness requiring referral or hospitalization, as determined by the health care provider, were excluded from participation. Given the high malaria transmission and parasite prevalence in the study location, it was expected that some participants would either become reinfected or continue to exhibit malaria during the study period and return to the health center/post. In such cases, the individual’s status as a returning participant was recorded, but all testing was repeated, and they were treated in the study analysis as a unique sample.

### Study procedures and RDT testing

Following enrollment, participants completed a brief questionnaire to collect information on demographics, current health status and medical history. Next, trained study staff collected capillary blood samples, performed the standard-of-care malaria RDT (the SD BIOLINE Malaria Ag Pf test; Abbott Diagnostics Korea Inc., Republic of Korea; catalog no. 05FK50) and conducted the three Rapigen RDTs under investigation: (i) BIOCREDIT Malaria Ag Pf (pLDH/HRPII) (catalog no. C13RHG25); (ii) BIOCREDIT Malaria Ag Pf (pLDH) (catalog no. C14RHG25); and (iii) BIOCREDIT Malaria Ag Pf/Pv (pLDH/pLDH) (catalog no. C61RHG25). All tests were conducted according to the manufacturer’s instructions and the results read within the specified timeframes. Test and control line intensities of the Rapigen test results were assigned by the reader based on comparison of test and control line observed intensities to 16-point (test) and 5-point (control) intensity scales on a card provided by Rapigen. All invalid test results were recorded. Results from the standard-of-care RDT were used to inform clinical care, and all patients who tested positive were treated according to national guidelines.

Next, 4 ml of venous blood was collected into an EDTA tube and transported under refrigerated conditions to the Institut Pasteur field station in Kédougou. At the field station, the venous blood was aliquoted, and the three BIOCREDIT RDTs were repeated. All test operators at the field station were blinded to the results from the RDTs performed at the clinic. One aliquot of venous blood was also used to prepare microscopy slides, and the remaining aliquots were frozen at − 20 °C. Microscopy slides were read by a trained microscopist at the Institut Pasteur de Dakar laboratory in Dakar, Senegal who was blinded to prior results. Frozen venous blood specimens were shipped on ice to PATH (Seattle, WA, USA) for reference PCR testing and confirmatory antigen concentration determination. Test operators performing the reference and confirmatory assays were blinded to all RDT and microscopy results and vice versa.

### Reference PCR testing by photoinduced electron transfer-PCR

Frozen whole blood in EDTA was analyzed for the detection of *Pf* nucleic acid using a PCR assay. A photoinduced electron transfer (PET)-PCR assay for *Pf* was conducted at PATH. For controls, the 3D7 *Pf* strain was used, and parasitemia was assessed by an expert microscopist who examined around 2000 erythrocytes to count the parasites. The erythrocyte count per microliter was determined using a Coulter counter, and the parasite density per microliter was calculated by multiplying the red blood cell (RBC) count by the parasitemia percentage. A twofold serial dilution, ranging from 2000, 1000, 500, 250, 125, 62.5, 31.25, 15.625, 7.8125 and 3.90625, to 1.953125 parasites/µl was created as controls in a standard curve. DNA was extracted from each dilution using 200 µl of sample. Amplification of *Pf* was performed by the PET-PCR assay as described by Lucchi et al. [29], and a cycle threshold (Ct) value < 40 was considered to be a positive result.

For patient samples, DNA was extracted from 100 µl of frozen whole blood using the QIAamp DNA Mini Kits (QIAGEN, Valencia, CA, USA; catalog no. 51106), eluted into 100 µl of elution buffer and stored at - 20 ºC. All samples were screened for *Pf* using forward (5′-ACC CCT CGC CTG GTG TTT TT-3′) and reverse (HEX-5′-agg cgg ata ccg cct ggT CGG GCC CCA AAA ATA GGA A-3′) self-quenching primers, as established by the Centers for Disease Control and Prevention [29]. The reaction was performed in a 20-μl reaction volume containing 2× TaqMan Environmental buffer 2.0 (Applied Biosystems, Grand Island, NY, USA; catalog no. 4398044), 62.5 nM each of the forward and reverse primer and 5 μl of template DNA. Cycling parameters were 95 ˚C for 10 min, followed by 45 cycles of denaturation at 95 ˚C for 10 s and annealing at 60˚C for 40 s. Ct values, defined as the number of cycles required for the fluorescent signal to cross the threshold (i.e. to exceed the background level), were recorded after the end of each annealing step. Specimens with Ct values < 40 were considered to be positive. Parasite densities of participant specimens were extrapolated from the standard curve. All assays were performed using Agilent Mx3005pro thermocyclers (Agilent Technologies, Santa Clara, CA, USA).

### Reference antigen concentration determination

Antigen concentration was determined with a quantitative antigen assay: Q-Plex™ Human Malaria Array (5-Plex) (Quansys Biosciences, Logan UT, USA). This is a chemiluminescent ELISA that allows the identification of malaria infection and the simultaneous detection of human C-reactive protein (CRP) in human blood, serum and plasma. This assay uses a quantitative sandwich enzyme immunoassay to measure malaria HRP2, PfLDH, PvLDH, Pan-specific LDH (PanLDH) antigens and human CRP in < 4 h [30, 31]. The cutoffs used for antigen positivity were as described previously [30].

### Usability study

Two usability studies were conducted to evaluate the *Pf-*only BIOCREDIT RDTs: the BIOCREDIT Malaria Ag Pf (pLDH) and the BIOCREDIT Malaria Ag Pf (pLDH/HRPII) RDTs. Participants included healthcare workers, who are representative of intended end users of malaria RDTs in rural and urban settings in Senegal. Each participant evaluated only one of the two RDTs and completed a questionnaire assessing label and packaging comprehension as well as interpretation of the results of images of RDTs [32].

### Data management and statistical analysis

All study results were recorded on data collection forms and double-entered into REDCap® (Research Electronic Data Capture, version 12.3.3), a web-based software platform with built-in validation rules to minimize data entry errors [33].

For the estimation of sample size, a 95% confidence interval (CI) was assumed, with an absolute precision of 0.025. In the absence of preliminary data, a 50% prevalence of *Pf* infection was assumed, yielding a target sample size of 241 participants.

A descriptive statistical analysis, including calculating point estimates, distributions and frequencies of responses, was conducted to summarize and characterize the study population. The number and percentage of participants infected with malaria, as determined by all assays, were assessed. Diagnostic accuracy was determined by calculating the sensitivity, specificity, positive predictive value (PPV) and negative predictive value (NPV) of all RDTs and microscopy in comparison to (i) the reference PCR assay and (ii) the quantitative antigen assay. The diagnostic accuracy of the quantitative antigen assay relative to the PCR reference method was also calculated. For comparisons using antigen concentrations as the reference method, the cognate target (PfLDH, HRP2 or both) was used to determine true positivity or negativity. All diagnostic performance results were reported with 95% CIs calculated using the conservative exact binomial method.

RDT performance was analyzed as a function of antigen concentration distribution and parasite density. This analysis was conducted only for the BIOCREDIT Malaria Ag Pf (pLDH/HRPII) RDT as it was the only test in this study with both PfLDH and HRP2 lines and had comparable PfLDH results to the other two BIOCREDIT products. As a first step, the relationships between HRP2 and PfLDH concentrations and parasite density, stratified by RDT result, were described using boxplots with jittered datapoints overlaid. Next, scatterplots of HRP2 versus PfLDH concentration stratified by RDT status and PCR status were used to visually explore the data. A sigmoidal dose response statistical model was then fitted within a Bayesian framework to estimate the relationship between antigen concentration and test operator-assigned RDT line intensity scores for each antigen. Finally, a logistic regression model was fitted within a Bayesian framework to estimate the relationship between antigen concentration and the probability of RDT positivity for each antigen [20]. In both models, antigen concentrations were log10 transformed to ensure appropriate scaling. Uninformative priors were used in the line intensity model. Both models were run for 10,000 iterations and convergence was assessed via visual checks of the coefficient trace plots.

The Rapigen BIOCREDIT Pf (pLDH/HRPII) test has previously been evaluated for analytical performance against antigen concentration using a standardized laboratory panel [20]. In that study, Golden et al. (2024) determined the test’s antigen LOD, defined as the concentration at which the test was expected to be positive 90% of the time, to be 525 pg/ml for HRP2 and 1318 pg/ml for LDH [20]. In the present study, we applied these cutoffs to predict the test result for each sample as detectable or not detectable by the RDT, depending on whether the measured cognate antigen concentration was above or below the LOD. The predicted clinical performance of the BIOCREDIT Malaria Ag Pf (pLDH/HRPII) RDT against both the reference PCR and antigen concentration results in this population was then calculated and compared against observed performance.

Statistical analysis was conducted using R version 4.2.1 (R Foundation for Statistical Computing, Vienna, Austria).

Usability assessment included both multiple-choice and open-ended questions, and responses were analyzed using descriptive statistics.

## Results

### Characteristics of the study population

A total of 236 participants were included in the study. Of these, 220 had venous blood available for reference assay analysis and were therefore included in the analytical sample. Of these 220 participants, capillary results for the investigational tests were not available for two participants, and one result from the BIOCREDIT Malaria Ag Pf (pLDH) test was excluded from analysis due to a data recording error. Microscopy results were available for 183 participants, and a subset of specimens from 200 participants with sufficient specimen volume underwent antigen concentration testing.

Table 1 presents the demographic information of the study participants. The majority of participants (60%) were aged > 16 years, and there was an even distribution of male (49%) and female (51%) participants. The study population reported a diverse range of symptoms, and 41% were febrile at enrollment, defined as having a body temperature of ≥ 37.5 °C.

**Table 1:** Demographic information of study participants

Demographics	Value	*N*	Proportion
Age (years)			
Mean (SD)	20.42 (14.81)	220	–
Range	0–71	220	–
*Age categories (years)*			
0–2	15	220	0.07
3–5	17	220	0.08
6–12	41	220	0.19
13–15	13	220	0.06
16–64	130	220	0.59
65+	4	220	0.02
*Sex*			
Male	107	220	0.49
Female	113	220	0.51
*Pregnancy status*			
Pregnant	9	113	0.08
Not pregnant	95	113	0.84
Rather not say	9	113	0.08
*Any antimalarial drug received in the last 4 weeks*			
Yes	55	219	0.25
No	164	219	0.75
*Symptom status*			
Abdominal pain	50	220	0.23
Chills	54	220	0.25
Conjunctival redness	2	220	0.01
Cough	43	220	0.20
Dizziness/vertigo	49	220	0.22
Dysuria	1	220	0.00
Fatigue	131	220	0.60
Fever (self-report)	173	220	0.79
Headache	151	220	0.69
Nausea	36	220	0.16
Stomachache	59	220	0.27
Sweats	13	220	0.06
Vomiting	72	220	0.33
Other	52	220	0.24
*Febrile at enrollment (temperature ≥ 37.5 °C)*			
Yes	89	219	0.41
No	130	219	0.59

SD Standard deviation

Of the 220 enrolled participants, 154 (70%) were *Pf*-positive by the reference PCR. For these PCR-confirmed *Pf-*positive cases, the geometric mean calculated parasite density was 12,607.74 parasites/µl, the mean HRP2 concentration was 3399.3 ng/ml and the mean PfLDH concentration was 556.6 ng/ml. No *Pv-*positive specimens were observed on any of the assays. One suspected *hrp2/hrp3* deletion case was identified in this population based on the criteria of being Pf-positive by PCR, with HRP2-negative and PfLDH-positive antigen concentration results. This specimen was negative for malaria by microscopy, and negative for the PvLDH antigen, but positive for the PanLDH antigen. No invalid results were obtained on any RDTs in this study.

### Diagnostic performance of investigational and comparator RDTs against reference PCR

Table 2 presents a summary of the diagnostic performance of all investigational and comparator tests compared to the PCR reference assay by specimen type. When the PCR reference method was used, microscopy showed the lowest overall performance with a sensitivity of 53% (95% CI 44.5–62.2). The BIOCREDIT Malaria Ag Pf (pLDH/HRPII) test showed the highest sensitivity of all evaluated tests at 78% (95% CI 70.9–84.5); the corresponding PPV and NPV were 94% (95% CI 88.9–97.7) and 64% (95% CI 53.5–73.9), respectively. The two BIOCREDIT PfLDH-only tests in this study showed the lowest performance. Both of these tests, as well as the PfLDH line alone on the BIOCREDIT Malaria Ag Pf (pLDH/HRPII) test, had lower sensitivity than the comparator HRP2-based RDT (71%; 95% CI 63.6–78.4). The BIOCREDIT Malaria Ag Pf (pLDH/HRPII) test also had the lowest specificity at 89% (95% CI 79.4–95.6), with the HRP2 line showing the lowest specificity.

**Table 2:** Diagnostic performance of investigational and comparator tests against reference PCR for detection of *Plasmodium falciparum*

Test	Target	Capillary					Venous				
		*N*	Sensitivity (95% CI)	Specificity (95% CI)	PPV (95% CI)	NPV (95% CI)	*N*	Sensitivity (95% CI)	Specificity (95% CI)	PPV (95% CI)	NPV (95% CI)
SD BIOLINE Ag Pf (#05FK50)	PfHRP2	220	0.714 (0.636–0.784)	0.939 (0.852–0.983)	0.965 (0.913–0.990)	0.585 (0.485–0.680)	N/A	N/A	N/A	N/A	N/A
BIOCREDIT Pf (pLDH/HRPII)	PfHRP2 and/or PfLDH positive	218	0.783 (0.709–0.846)	0.894 (0.794–0.956)	0.944 (0.889–0.977)	0.641 (0.535–0.739)	220	0.818 (0.748–0.876)	0.894 (0.794–0.956)	0.947 (0.895–0.979)	0.678 (0.569–0.774)
	PfHRP2 only	218	0.776 (0.702–0.840)	0.924 (0.832–0.975)	0.959 (0.908–0.987)	0.642 (0.537–0.738)	220	0.812 (0.741–0.870)	0.924 (0.832–0.975)	0.962 (0.913–0.987)	0.678 (0.571–0.772)
	PfLDH only	218	0.618 (0.536–0.696)	0.955 (0.873–0.991)	0.969 (0.912–0.994)	0.521 (0.428–0.612)	220	0.662 (0.582–0.736)	0.970 (0.895–0.996)	0.981 (0.932–0.998)	0.552 (0.457–0.644)
BIOCREDIT Pf/Pv (pLDH/pLDH)	PfLDH	218	0.645 (0.563–0.721)	0.955 (0.873–0.991)	0.970 (0.916–0.994)	0.538 (0.444–0.631)	220	0.662 (0.582–0.736)	0.970 (0.895–0.996)	0.981 (0.932–0.998)	0.552 (0.457–0.644)
BIOCREDIT Pf (pLDH)	PfLDH	217	0.642 (0.560–0.719)	0.955 (0.873–0.991)	0.970 (0.915–0.994)	0.538 (0.444–0.631)	220	0.662 (0.582–0.736)	0.970 (0.895–0.996)	0.981 (0.932–0.998)	0.552 (0.457–0.644)
Microscopy	Parasites	N/A	N/A	N/A	N/A	N/A	183	0.534 (0.445–0.622)	0.981 (0.897–1.000)	0.986 (0.924–1.000)	0.455 (0.361–0.552)

*Ag* Antigen,* CI* confidence interval,* HRPII/2* histidine-rich protein 2,* N/A* not applicable,* NPV* negative predictive value, *Pf* *Plasmodium falciparum*,* PfHRP2 Plasmodium falciparum-*specific histidine-rich protein 2,* PfLDH* *Plasmodium falciparum-*specific lactate dehydrogenase,* pLDH* *Plasmodium* lactate dehydrogenase,* PPV* positive predictive value, *Pv* *Plasmodium vivax*

### Diagnostic performance of investigational and comparator RDTs against antigen concentration

Of the 200 participants whose samples were tested with the quantitative antigen assay, 66% (131) of were *Pf*-positive. Table 3 presents a summary of the diagnostic performance of all investigational and comparator tests, compared to the quantitative antigen assay, by specimen type. When RDT performance was compared against the quantitative antigen assay as the reference method, the sensitivity of all tests on both specimen types was significantly higher than when compared against the PCR reference, with the largest improvements observed on the PfLDH test lines. The same trend was observed for specificity, with the exception of the HRP2 line on the BIOCREDIT Malaria Ag Pf (pLDH/HRPII) test with capillary specimens, which showed 92% specificity (95% CI 82.5–96.8), as compared to 92% (95% CI 83.2–97.5) when compared against PCR. Overall, the PfLDH-only tests had the highest performance compared to the antigen assay, irrespective of specimen type. This can be understood in the context of the performance of the quantitative antigen assay relative to the reference PCR method, which also showed the lowest sensitivity (71%) for the detection of PfLDH (Additional file 1: Table S1). All RDTs performed slightly better on venous blood specimens than on capillary specimens using both reference methods, although differences were negligible and within the 95% CIs.

**Table 3:** Diagnostic performance of investigational and comparator tests against the reference quantitative antigen assay for *P. falciparum*

Test	Target	Capillary blood specimens					Venous blood specimens				
		*N*	Sensitivity (95% CI)	Specificity (95% CI)	PPV (95% CI)	NPV (95% CI)	*N*	Sensitivity (95% CI)	Specificity (95% CI)	PPV (95% CI)	NPV (95% CI)
SD BIOLINE Ag Pf (#05FK50)	PfHRP2	200	0.760 (0.677–0.831)	0.958 (0.881–0.991)	0.970 (0.916–0.994)	0.687 (0.586–0.776)	N/A	N/A	N/A	N/A	N/A
BIOCREDIT Pf (pLDH/HRPII)	PfHRP2 and/or PfLDH positive	198	0.837 (0.762–0.896)	0.928 (0.839–0.976)	0.956 (0.900–0.985)	0.753 (0.647–0.840)	200	0.893 (0.827–0.940)	0.971 (0.899–0.996)	0.983 (0.941–0.998)	0.827 (0.727–0.902)
	PfHRP2 only	198	0.819 (0.741–0.882)	0.915 (0.825–0.968)	0.945 (0.885–0.980)	0.739 (0.634–0.827)	200	0.876 (0.806–0.927)	0.958 (0.881–0.991)	0.974 (0.926–0.995)	0.810 (0.709–0.887)
	PfLDH only	198	0.869 (0.786–0.928)	0.990 (0.945–1.000)	0.989 (0.938–1.000)	0.883 (0.808–0.936)	200	0.911 (0.838–0.958)	0.990 (0.945–1.000)	0.989 (0.942–1.000)	0.916 (0.846–0.961)
BIOCREDIT Pf/Pv (pLDH/pLDH)	PfLDH	198	0.889 (0.810–0.943)	0.980 (0.929–0.998)	0.978 (0.922–0.997)	0.898 (0.825–0.948)	200	0.911 (0.838–0.958)	1.000 (0.963–1.000)	1.000 (0.961–1.000)	0.917 (0.848–0.961)
BIOCREDIT Pf (pLDH)	PfLDH	197	0.898 (0.820–0.950)	0.990 (0.945–1.000)	0.989 (0.939–1.000)	0.907 (0.836–0.955)	200	0.911 (0.838–0.958)	1.000 (0.963–1.000)	1.000 (0.961–1.000)	0.917 (0.848–0.961)

*Ag* Antigen,* CI* confidence interval,* HRPII/2* histidine-rich protein 2,* N/A* not applicable,* NPV* negative predictive value, *Pf* *Plasmodium falciparum*,* PfHRP2 Plasmodium falciparum-*specific histidine-rich protein 2,* PfLDH* *Plasmodium falciparum-*specific lactate dehydrogenase,* pLDH* *Plasmodium* lactate dehydrogenase,* PPV* positive predictive value, *Pv* *Plasmodium vivax*

### Antigen concentration distribution and performance of the BIOCREDIT Malaria Ag (pLDH/HRPII) RDT

**Fig. 1:**
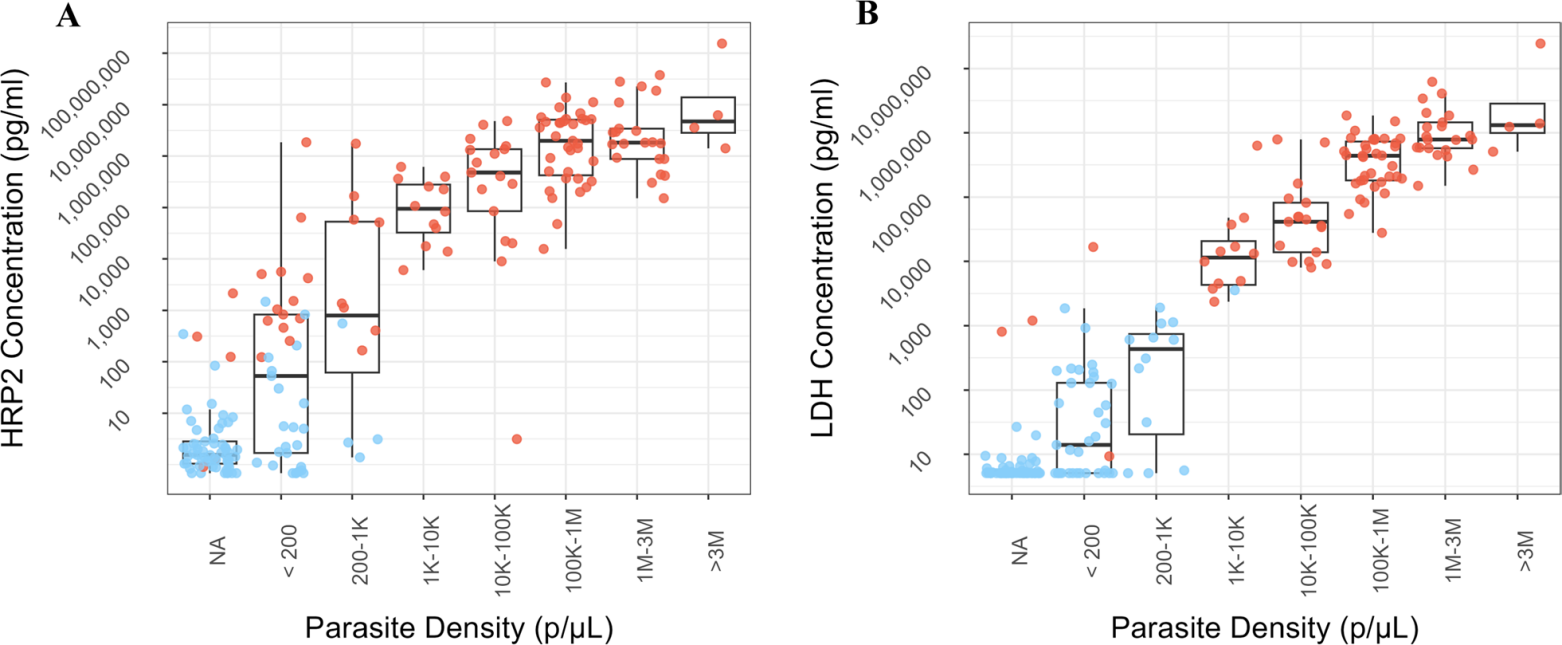
Box plots of HRP2 (**A**) and *Plasmodium falciparum-*specific lactate dehydrogenase (PfLDH) (**B**) antigen concentration distributions as a function of parasite density in PCR-confirmed cases. Results by line from the BIOCREDIT Pf (pLDH/HRPII) test are indicated in color, with positive test lines shown in red and negative test lines shown in blue for the HRP2 line and PfLDH line in panels **A** and **B**, respectively. HRP2, Histidine-rich protein 2; LDH, lactate dehydrogenase; NA, not applicable

Figure 1 summarizes the relationship between HRP2 concentration and parasite density (Fig. 1a) and LDH concentration and parasite density (Fig. 1b), with color coding used to distinguish the results for each line on the BIOCREDIT Malaria Ag Pf (pLDH/HRPII). The HRP2 line detected specimens with lower target antigen concentrations compared to the PfLDH line.

The BIOCREDIT Malaria Ag Pf (pLDH/HRPII) RDT had a 50% probability of detecting HRP2 at an antigen concentration of 0.175 ng/ml, and a 90% probability at 2.84 ng/ml (Additional file 2: Figure S1). The 50% and 90% probabilities of detection for PfLDH were 2.17 and 12.24 ng/ml, respectively (Additional file 2: Figure S1.

**Fig. 2:**
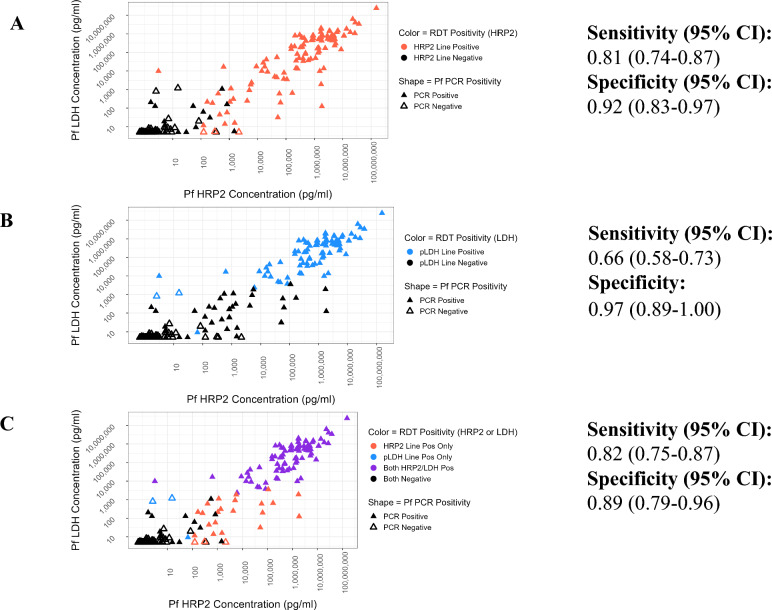
HRP2 concentration vs PfLDH in clinical specimens. **A**, **B** results for the HRP2 (**A**) and PfLDH (**B**) test lines on the RDT for each clinical specimen.** C** RDT results for both test lines. Filled triangles denote PCR-confirmed *Plasmodium falciparum* specimens, open triangles denote PCR-negative specimens. Test line results from the BIOCREDIT Pf (pLDH/HRPII) test are indicated in color, with HRP2-positive specimens shown in red, LDH-positive specimens shown in blue (**B**), and specimens positive on both lines shown in purple (**C**). CI, Confidence interval; HRP2, histidine-rich protein 2; LDH, lactate dehydrogenase; Pf, *Plasmodium falciparum*; PfHRP2,* Plasmodium falciparum-*specific histidine-rich protein 2; PfLDH, *Plasmodium falciparum-*specific lactate dehydrogenase; pLDH, *Plasmodium* lactate dehydrogenase; RDT, rapid diagnostic test

Figure 2 shows the contribution of each test line to the overall performance of the BIOCREDIT Malaria Ag Pf (pLDH/HRPII) test, displaying the PfLDH and HRP2 concentrations for each clinical specimen. Figure  2a and 2b shows the results for the HRP2 and PfLDH test lines, respectively, on the RDT for each clinical specimen; the results combined are shown in Fig. 2c. More PCR-confirmed specimens had detectable HRP2 concentrations on the HRP2 line but not the LDH line, compared to specimens that were detectable on the LDH line but not the HRP2 line (Fig. 2c).

**Fig. 3:**
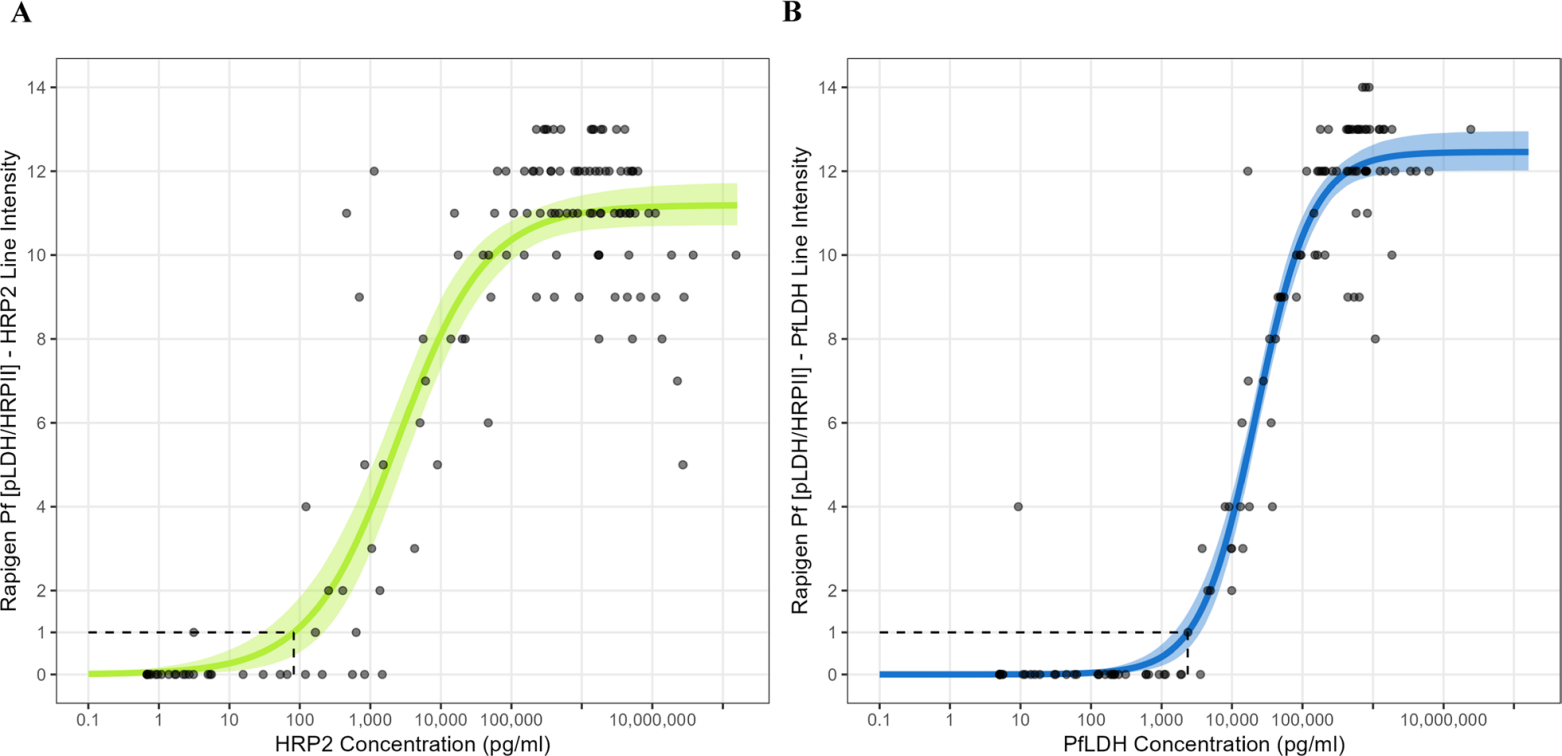
Correlation between antigen concentration and the BIOCREDIT Pf (pLDH/HRPII) RDT line intensity. 0 is a negative test result, 1–15 are positive test results.** A** The intensity of the HRP2 line on the RDT plotted against the HRP2 concentration,** B** the intensity of the LDH line on the RDT plotted against the LDH concentration. Dotted lines indicate the concentration at which line intensity is weakly visible (score of 1). HRP2, histidine-rich protein 2; LDH, lactate dehydrogenase; Pf, *Plasmodium falciparum*; PfLDH, *Plasmodium falciparum-*specific lactate dehydrogenase; pLDH, *Plasmodium* lactate dehydrogenase; RDT, rapid diagnostic test

RDT line intensity was also correlated with the antigen concentration for the BIOCREDIT Pf (pLDH/HRPII) test (Fig. 3). The HRP2 line showed an antigen-dependent increase in visible intensity of between approximately 0.1 and 100 ng/ml (100–100,000 pg/ml). The PfLDH line showed an antigen-dependent increase in visible intensity of between approximately 2 and 100 ng/ml (2000–100,000 pg/ml). For both lines, the test lines for specimens with HRP2 or PfLDH concentrations > 100 ng/ml appeared to be saturated and did not significantly increase with higher antigen concentrations.

### Performance prediction based on analytical sensitivity

Table 4 shows the predicted sensitivities based on the test’s analytical LOD as applied to this study population against both the quantitative antigen and PCR reference methods and the observed sensitivities (also shown in Tables 2, 3) for each antigen on the RDT and the combined RDT test result. The specificity against the quantitative antigen reference method is, by definition, 100% for all antigens; therefore, only specificity against PCR is presented. Overall, the predicted and observed sensitivities were comparable. When using the antigen concentration reference method, the predicted sensitivity of the RDT was slightly lower than the observed sensitivity for the detection of HRP2 (84% vs 88%) but slightly higher for the detection of PfLDH (92% vs 91%). For the detection of either HRP2 and/or PfLDH, the predicted sensitivity was lower than the observed sensitivity (84% vs 89%). Against the PCR reference method, the test’s predicted sensitivity was lower than the observed sensitivity for HRP2 (78% vs 81%) and slightly higher for PfLDH (67% vs 66%). For HRP2 and/or PfLDH, the predicted sensitivity was, again, lower (78% vs 82%).

**Table 4:** Performance of the Rapigen BIOCREDIT Pf (pLDH/HRPII) rapid diagnostic test

Test line	Quantitative antigen assay reference		PCR reference			
	Sensitivity (95% CI)^a^		Sensitivity (95% CI)^a^		Specificity (95% CI)^b^	
	Predicted	Observed (Table 3)	Predicted	Observed (Table 2)	Predicted	Observed (Table 2)
HRP2 line	0.844 (0.769–0.902)	0.876 (0.806–0.927)	0.775 (0.697–0.842)	0.812 (0.741–0.870)	0.984 (0.912–1.000)	0.958 (0.881–0.991)
PfLDH line	0.920 (0.848–0.965)	0.911 (0.838–0.958)	0.667 (0.581–0.745)	0.662 (0.582–0.736)	1.000 (0.941–1.000)	0.990 (0.945–1.000)
HRP2 and/or PfLDH lines	0.838 (0.764–0.897)	0.893 (0.827–0.940)	0.783 (0.704–0.848)	0.818 (0.748–0.876)	0.984 (0.912–1.000)	0.971 (0.899–0.996)

The table presents (i) the predicted results of the test based on its analytical limit of detection, and (ii) the observed results of the test against each reference method

*CI* Confidence interval,* HRP2/HRPII* histidine-rich protein 2,* PfLDH Plasmodium falciparum*-specific lactate dehydrogenase,* pLDH*: Plasmodium lactate dehydrogenase

^a^Sensitivity is presented against confirmed positive cases separately by the quantitative antigen and PCR reference assays

^b^Specificity is presented against confirmed negative cases by PCR

For overall test performance against PCR, the HRP2 line was predicted to contribute the most to test sensitivity, with a marginal increase when the LDH line was included. Indeed, this pattern of performance was observed in the study. Additionally, the HRP2 line was predicted to contribute to a drop in the specificity of the test compared to LDH line when using the PCR reference method, as was observed in the study.

### Usability results

In total, ten healthcare workers evaluated the usability of the BIOCREDIT Pf (pLDH) test, and 16 evaluated the BIOCREDIT Pf (pLDH/HRPII) test. All participants successfully conducted the tests, and no critical errors were observed during the use of either test. Responses to the multiple-choice label comprehension questionnaires indicated that most participants understood the tests’ intended uses, safety information, and warnings (Additional File 3: Table S2). The greatest source of error in the test’s workflow was in identifying the correct number of assay buffer drops to add to the test devices and determining the correct reading window for test result interpretation. Additionally, most contrived test result images were correctly read and interpreted by participants, with an overall interpretation error rate of 4.0% for the BIOCREDIT Pf (pLDH) test and 4.8% for the BIOCREDIT Pf (pLDH/HRPII) test (Additional file 4: Table S3). Ninety percent of participants evaluating the BIOCREDIT Pf (pLDH) test reported that it was either “easy” or “very easy” to use, compared to 70% of those evaluating the BIOCREDIT Pf (pLDH/HRPII) test.

## Discussion

This study evaluated the performance of three novel LDH-based malaria RDTs in a febrile population in Kédougou, Senegal, and assessed RDT performance as a function of antigen concentration distribution. Improved sensitivity for PfLDH is critical for RDTs to detect emerging *hrp2/3* deletions. The WHO recommends switching to LDH-based RDTs only when there is a confirmed significant prevalence (> 5%) of false-negative RDT results arising from *hrp2/hrp3* deletions due to the documented lower sensitivity of LDH-based RDTs for* Pf* [16]. This study population had only one specimen with a suspected deletion based on the HRP2 and PfLDH results from the antigen quantification assay; however, this case could also be attributed to an infection from *Plasmodium malariae* or *Plasmodium ovale*, which have previously been found in this area of Senegal [27]. Specimens in this study were not genotyped for *hrp2/hrp3* deletions.

Among all RDTs included in this study, the BIOCREDIT Malaria Ag Pf (pLDH/HRPII) test, which has test lines for both HRP2 and PfLDH, showed the highest sensitivity for the detection of* Pf* at 78%. This sensitivity is slightly higher than that of the SD BIOLINE HRP2-based RDT (71%), an established and WHO-prequalified product currently in routine use in Senegal. Although the HRP2 line alone on the BIOCREDIT Malaria Ag Pf (pLDH/HRPII) test was more sensitive than the comparator SD BIOLINE HRP2-based test (78% vs 71% on capillary specimens), the sensitivities of the PfLDH lines alone on all three BIOCREDIT RDTs (61–64% on capillary specimens) were lower than that of the HRP2-based comparator. This result suggests that in populations such as the one included in the present study, where *hrp2/hrp3* deletions are uncommon, the PfLDH line alone cannot compensate for the performance of the HRP2 line, even with the improved LOD for LDH on the Rapigen tests. Further improvement in the sensitivity of PfLDH detection by RDTs would support more robust clinical diagnosis of *Pf* malaria in populations with varied underlying prevalences of *hrp2/hrp3* deletions. In the meantime, to achieve the best sensitivity for detection of all* Pf* cases, RDTs should detect both HRP2 and PfLDH, possibly combined on the same line to streamline usability with a single line* Pf* result.

On the BIOCREDIT Malaria Ag Pf (pLDH/HRPII) RDT, a higher rate of false positives was observed on both capillary and venous specimens when considering results based on either test line, leading to a consequently lower specificity than that of the other RDTs included in this evaluation. For most of these specimens (5/7), the measured antigen concentrations were below the detection levels or lower than the LOD of the RDT. Some drop in specificity may be attributed to the persistence of HRP2, as shown in the predicted performance of the RDT compared to the PCR reference assay, in contrast to LDH, where the presence of LDH closely corresponds to the presence of parasite DNA.

The results of this study are consistent with findings from other clinical evaluations of these tests. In Burundi, Niyukuri et al*.* [21] reported sensitivities of 79.9% and 72.3% for the BIOCREDIT Malaria Ag Pf (pLDH/HRPII) test in clinical and subclinical populations, respectively, in a sample with only two *hrp3* deletions [21]. These authors also similarly reported lower specificity for this test compared to the HRP2-only comparator RDT in both clinical and subclinical cases (82.4% vs 96.2% for clinical cases; 84.4% vs 93.4% for subclinical cases). Another study conducted in Djibouti, where there are high rates of *hrp2/hrp3* deletions and* Pv* cases [34–36], found a sensitivity and specificity of 88.2% and 100%, respectively, on the BIOCREDIT Malaria Ag Pf/Pv (pLDH/pLDH) for the detection of* Pf* [23].

The inclusion of a quantitative assay to determine antigen concentration in the present study allowed for assessment of RDT performance relative to the cognate analytes detected by each RDT test line. The performance of the RDT, evaluated against a reference assay for the same analyte (in contrast to microscopy or PCR), demonstrated improved sensitivity and specificity for both antigens across all RDTs. The relative sensitivities of the individual antigen test lines, when using the quantitative antigen reference assay compared to PCR/DNA as a reference standard, are driven by the analytical sensitivity of the reference assay for the analyte, the overall antigen concentration distribution in the specimens and the absolute LODs of the RDTs for the antigens. The quantitative antigen assay has a higher clinical LOD for LDH compared to HRP2 [30], which results in missing clinical samples with low LDH concentrations. Additionally, clinical specimens overall have lower LDH concentrations compared to HRP2, resulting in fewer PCR samples with measurable LDH levels, as has been shown previously [30, 31]. Consequently, the sensitivity of the antigen lines on the RDTs is highest for LDH when using the antigen reference method but lowest when using the PCR method. Most essentially, the lower analytical sensitivity of the LDH lines combined with the lower abundance of LDH in clinical specimens compared to HRP2 results in a lower sensitivity of the LDH lines compared to the HRP2 lines in the RDTs evaluated in this study.

Although not an intended endpoint of the study, the training of test operators to record RDT line intensity in this study enabled the demonstration that—even by eye—a reasonable relationship was present between RDT line intensity and antigen concentration. Given that results from challenge and longitudinal studies suggest that the ratio of HRP2 to LDH can be used to differentiate active from recently cleared infections [37], further investigation into the utility of HRP2/LDH line intensities from an RDT for this purpose is warranted.

The analytical sensitivity (LOD) of the BIOCREDIT Malaria Ag Pf (pLDH/HRPII) RDT was previously determined through laboratory-based benchmarking studies [20]. In this study, the laboratory-derived LOD of the BIOCREDIT Malaria Ag Pf (pLDH/HRPII) test was combined with the distribution of antigen concentrations and reference PCR results from the study population to estimate the predicted performance of the RDT, which was then compared to the observed performance from this study. Overall, the predicted performance of the test was comparable to the observed performance, demonstrating the value of understanding the underlying antigen concentration in a population and validating the ability to predict RDT performance based on analytical LODs. Notably, as we observed, the predictions confirmed HRP2 as the largest driver of clinical sensitivity for the test in this population, with LDH contributing to a marginal improvement in clinical sensitivity for* Pf* infection. Additionally, as observed, there is a larger drop in specificity due to the HRP2 line in contrast to the LDH line. This predicted drop in specificity arises exclusively from specimens which have antigen but are PCR negative. The drops in specificity are driven either by the emergence of antigen in advance of DNA, or more likely, by the accumulation and persistence of antigen after DNA clearance. Other sources of cross-reactivity not accounted for in analytical studies are likely to contribute to the actual observed clinical specificity.

Using analytical performance to predict the performance of new RDTs in different populations with varying parasite density distributions is a valuable tool for assessing the impact of new tests in diverse contexts of use. As previously shown for SARS-CoV-2 RDTs, where quantitative N-antigen data could be used to predict clinical sensitivity across different specimen types [38, 39], understanding the underlying HRP2 and LDH concentration distributions across various use cases and epidemiological settings (e.g. case management, asymptomatic, pregnancy, low and high transmission) can improve predictions of test performance in these contexts.

From a usability perspective, the present study demonstrated that intended users in this setting were able to accurately comprehend key elements of the product labels and correctly interpret images of results. This aligns with a long history of prior research demonstrating the simplicity of these tests and their ability to be conducted by end users [40–42]. The most common errors related to the correct identification of the number of assay buffer drops to add to the test devices and determining the appropriate reading window for interpreting test results. These aspects of malaria RDT workflows often vary between products, highlighting the importance of context-specific training that emphasizes key aspects of the test procedure [43]. The results suggest a slight user preference for the *Pf* RDT with a single test line compared to the product with two separate test lines for HRP2 and PfLDH. However, both products were evaluated by small samples and two different groups of users; therefore, comparisons should be interpreted with caution.

Several other limitations of this study should also be noted. Microscopy and antigen quantification results were only available for a subset of participants. Although* Pv* has been observed in Eastern Senegal [13, 27, 44, 45], no cases were identified in this population on any of the tests conducted in this study. Consequently, the performance of the BIOCREDIT Malaria Ag Pf/Pv (pLDH/pLDH) test for the detection of *Pv* malaria was not assessed, and test performance on specimens with mixed infections could not be evaluated. Lastly, this study assessed test performance in a symptomatic, febrile population in an area of high endemicity. Analytical benchmarking and prior evaluations suggest that relative performance improvements may be expected with asymptomatic populations and subclinical cases [20, 21, 24]. Future studies should further investigate test performance in these contexts.

## Conclusions

In summary, this study confirms that despite the higher analytical sensitivity of the BIOCREDIT tests’ LDH line compared to other currently WHO-prequalified RDTs, the HRP2 line primarily drives the sensitivity of the RDT in this high-burden setting with negligible suspected *hrp2/hrp3* deletions. An RDT that performs equally to support clinical diagnosis of *Pf* malaria, regardless of the underlying prevalence of *hrp2/hrp3* deletions, should detect both HRP2 and LDH, possibly on the same line to simplify interpretation.

## Supplementary Information

Additional file 1: Table S1. Diagnostic performance of the quantitative antigen concentration assay against the reference PCR for the detection of *P. falciparum.*

Additional file 2: Figure S1. Probability of test line positivity on the BIOCREDIT Pf (pLDH/HRPII) RDT as a function of antigen concentration. In panel A, the probability of the HRP2 test line positivity is plotted against HRP2 concentration. In panel B, the probability of the LDH test line positivity is plotted against LDH concentration. The 50% and 90% probabilities of positivity are shown on both graphs.

Additional file 3: Table S2. Label comprehension questionnaire results for the Pf (pLDH) and Pf (pLDH/HRPII) tests.

Additional file 4: Table S3. Result interpretation questionnaire results for (a) the Pf (pLDH) test and (b) the Pf (pLDH/HRPII) test.

## Data Availability

The dataset generated and analyzed during the current study is available in the Harvard Dataverse repository, at https://doi.org/10.7910/DVN/0OMVWZ.

## Abbreviations

Ag: Antigen
CNERS: Comité National d’Ethique pour la Recherche en Santé
CRP: C-reactive protein
HRPII/HRP2: Histidine-rich protein 2
LDH: Lactate dehydrogenase
LOD: Limit of detection
PET: Photoinduced electron transfer
*Pf*: *Plasmodium falciparum*
PfLDH: *Plasmodium falciparum-*specific lactate dehydrogenase
pLDH: *Plasmodium* lactate dehydrogenase
PPV: Positive predictive value
*Pv*: *Plasmodium vivax*
NPV: Negative predictive value
RDT: Rapid diagnostic test
WCG: WIRB-Copernicus Group
WHO: World Health Organization
