# Supplementary figures and images for: Performance and usability evaluation of three LDH-based malaria rapid diagnostic tests in Kédougou, Senegal
Source: Parasit Vectors. 2025 Jul 12;18:280. doi: 10.1186/s13071-025-06914-9 (PMC12255971; doi:10.1186/s13071-025-06914-9)

## Slide 1
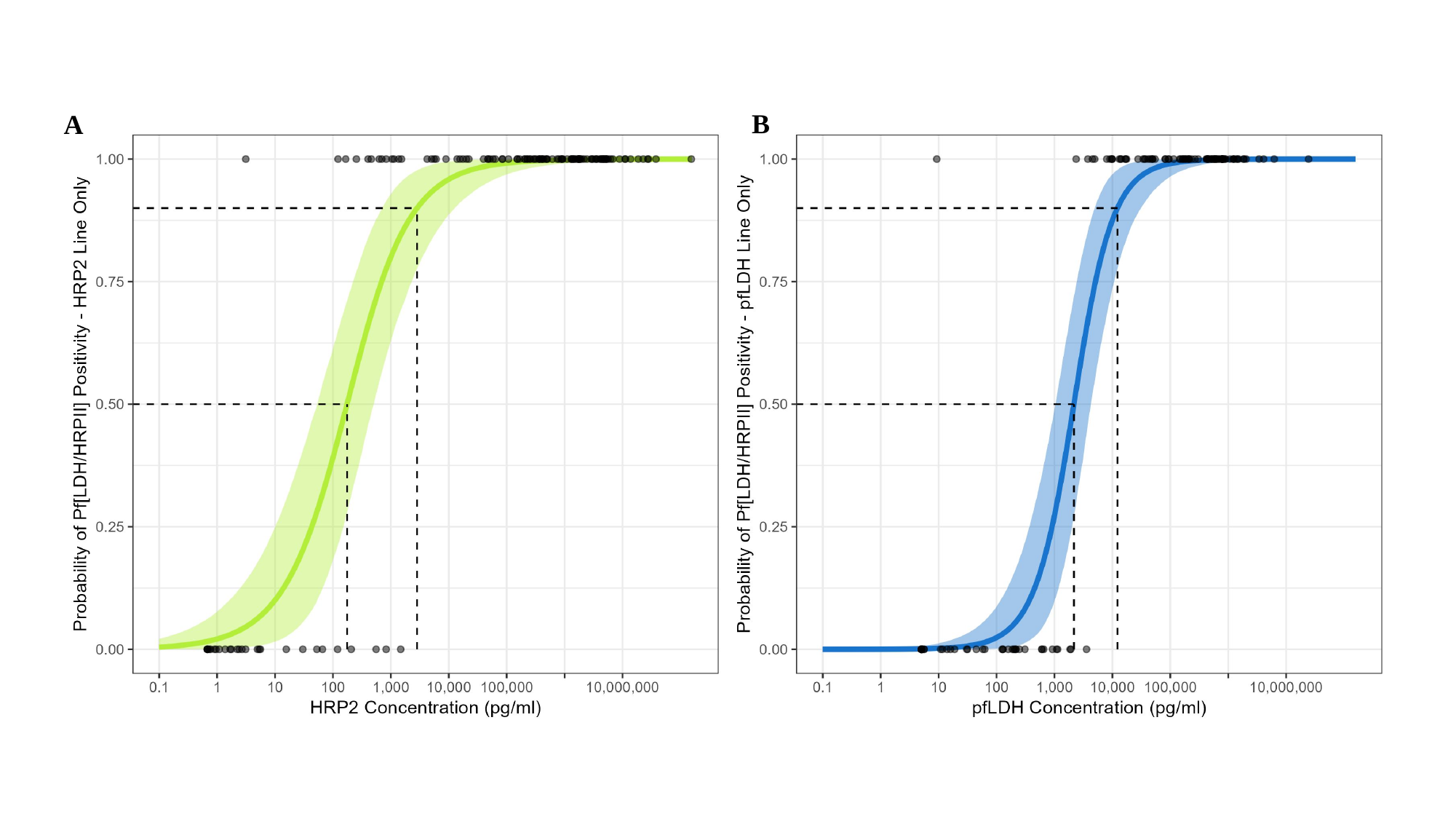

B
A

Supplement: Supplementary file 1 — Additional file 1: Table S1. Diagnostic performance of the quantitative antigen concentration assay against the reference PCR for the detection of P. falciparum. [file 13071_2025_6914_MOESM1_ESM.pptx]
