# Supplementary material for: Performance and usability evaluation of three LDH-based malaria rapid diagnostic tests in Kédougou, Senegal
Source: Parasit Vectors. 2025 Jul 12;18:280. doi: 10.1186/s13071-025-06914-9 (PMC12255971; doi:10.1186/s13071-025-06914-9)
Supplement: Supplementary file 2 — Additional file 2: Figure S1. Probability of test line positivity on the BIOCREDIT Pf (pLDH/HRPII) RDT as a function of antigen concentration. In panel A, the probability of the HRP2 test line positivity is plotted against HRP2 concentration. In panel B, the probability of the LDH test line positivity is plotted against LDH concentration. The 50% and 90% probabilities of positivity are shown on both graphs. [file 13071_2025_6914_MOESM2_ESM.docx]

**Supplementary Table 1.** Diagnostic performance of the quantitative antigen concentration assay against the reference PCR for the detection of *P. falciparum.*

| **Test** | **Target** | **Venous** | | | | |
| --- | --- | --- | --- | --- | --- | --- |
|  |  | *N* | *Sensitivity*  *(95% CI)* | *Specificity*  *(95% CI)* | *PPV*  *(95% CI)* | *NPV*  *(95% CI)* |
| Quantitative antigen assay | PfHRP2 | 199 | 0.855  (0.785 – 0.909) | 0.836  (0.719 – 0.918) | 0.922  (0.861 – 0.962) | 0.718  (0.599 – 0.819) |
|  | PfLDH | 199 | 0.710  (0.627 – 0.784) | 0.967  (0.887 – 0.996) | 0.980  (0.930 – 0.998) | 0.596  (0.493 – 0.693) |
|  | Pf (HRP2 and/or PfLDH positive | 199 | 0.862  (0.793 – 0.915) | 0.820  (0.700 – 0.906) | 0.915  (0.854 – 0.957) | 0.725  (0.604 – 0.825) |

*Abbreviations:* CI, confidence interval; HRP2, histidine-rich protein 2; PCR, polymerase chain reaction; PfLDH, *Plasmodium falciparum-*specific lactate dehydrogenase; PPV, positive predictive value; NPV, negative predictive value
