# Supplementary material for: Performance and usability evaluation of three LDH-based malaria rapid diagnostic tests in Kédougou, Senegal
Source: Parasit Vectors. 2025 Jul 12;18:280. doi: 10.1186/s13071-025-06914-9 (PMC12255971; doi:10.1186/s13071-025-06914-9)
Supplement: Supplementary file 3 — Additional file 3: Table S2. Label comprehension questionnaire results for the Pf (pLDH) and Pf (pLDH/HRPII) tests. [file 13071_2025_6914_MOESM3_ESM.docx]

**Supplementary Table 2.** Label comprehension questionnaire results for the Pf (pLDH) and Pf (pLDH/HRPII) tests.

| Question | Correct responses  **n (%)** | |
| --- | --- | --- |
|  | BIOCREDIT  Pf (pLDH) Test | BIOCREDIT Pf (pLDH/HRPII) Test |
| True or false: the test can be used to detect infection with *Pf* parasite causing malaria in humans. | 10 (100.0%) | 16 (100.0%) |
| What does the test measure? | 10 (100.0%) | 13 (81.3%) |
| Which plasmodium antigen can be detected using the test? | 9 (90.0%) | 15 (93.8%) |
| The test can be used with which type(s) of samples? | 7 (70.0%) | 16 (100.0%) |
| At what temperature should the test kit be stored? | 10 (100.0%) | 15 (93.8%) |
| When should you apply the assay buffer to the test device? | 10 (100.0%) | 16 (100.0%) |
| How much blood is required to run the test? | 7 (70.0%) | 12 (75.0%) |
| How many drops of assay buffer should you add to the test device? | 5 (50.0%) | 10 (62.5%) |
| How many test lines can you see on the device (including control line)? | 9 (90.0%) | 16 (100.0%) |
| How long should you wait to interpret the test results? | 10 (100.0%) | 16 (100.0%) |
| True or false: You can read the test result after 35 minutes. | 7 (70.0%) | 8 (50.0%) |
| True or false: with the presence of any test line, no matter how faint, the result is considered positive | 8 (80.0%) | 15 (93.8%) |
| Can you re-use the test device? | 10 (100.0%) | 16 (100.0%) |
| Where should the blood sample be applied to the test device? | 10 (100.0%) | 16 (100.0%) |
| Where should the assay buffer be applied to the test device? | 10 (100.0%) | 16 (100.0%) |

| **Key** |
| --- |
| >85% |
| 70%-85% |
| <70% |
