# Supplementary material for: Performance and usability evaluation of three LDH-based malaria rapid diagnostic tests in Kédougou, Senegal
Source: Parasit Vectors. 2025 Jul 12;18:280. doi: 10.1186/s13071-025-06914-9 (PMC12255971; doi:10.1186/s13071-025-06914-9)
Supplement: Supplementary file 4 — Additional file 4: Table S3. Result interpretation questionnaire results for (a) the Pf (pLDH) test and (b) the Pf (pLDH/HRPII) test. [file 13071_2025_6914_MOESM4_ESM.docx]

**Supplementary Table 3.** Result interpretation questionnaire results for (a) the Pf (pLDH) test, and (b) the Pf (pLDH/HRPII) test.

A) BIOCREDIT Pf (pLDH) test

| Image code | Control | Test Line | Interpretation | Correct responses  n (%) | Incorrect responses selected (n) |
| --- | --- | --- | --- | --- | --- |
| A | Visible | Strong | Positive | 10 (100.0%) |  |
| B | None | Strong | Invalid | 9 (90.0%) | Positive (1) |
| C | Visible | Weak | Positive | 10 (10.0%) |  |
| D | Visible | None | Negative | 9 (90.0%) | Invalid (1) |
| E | None | None | Invalid | 10 (100.0%) |  |

B) BIOCREDIT Pf (pLDH/HRPII) test

| Image code | Control | HRPII Line | pLDH Line | Interpretation | Correct responses  n (%) | Incorrect responses selected (n) |
| --- | --- | --- | --- | --- | --- | --- |
| A | None | None | None | Invalid | 16 (100.0%) |  |
| B | Visible | Weak | Strong | Positive | 16 (100.0%) |  |
| C | Visible | Strong | Strong | Positive | 15 (93.8%) | Negative (1) |
| D | Visible | Weak | Weak | Positive | 15 (93.8%) | Negative (1) |
| E | None | Strong | Weak | Invalid | 15 (93.8%) | Negative (1) |
| F | Visible | Weak | None | Positive | 13 (81.3%) | Negative (3) |
| G | Visible | None | Strong | Positive | 15 (93.8%) | Negative (1) |
| H | Visible | None | Weak | Positive | 16 (100.0%) |  |
| I | Visible | None | None | Negative | 16 (100.0%) |  |
| J | Visible | Strong | None | Positive | 15 (93.8%) | Negative (1) |
| K | None | Weak | None | Invalid | 15 (93.8%) | Negative (1) |
| L | None | None | Weak | Invalid | 15 (93.8%) | Negative (1) |
| M | Visible | Strong | Weak | Positive | 16 (100.0%) |  |

| **Key** |
| --- |
| >85% |
| 70%-85% |
| <70% |
